# The Others Are Too Loud! Children’s Experiences and Thoughts Related to Voice, Noise, and Communication in Nordic Preschools

**DOI:** 10.3389/fpsyg.2019.01954

**Published:** 2019-08-21

**Authors:** Anita McAllister, Leena Rantala, Valdís Ingibjörg Jónsdóttir

**Affiliations:** ^1^CLINTEC, Division of Speech and Language Pathology, Karolinska Institutet, Solna, Sweden; ^2^Functional Area Speech and Language Pathology, Karolinska University Hospital, Stockholm, Sweden; ^3^Department of Logopedics, University of Tampere, Tampere, Finland; ^4^“Thad er malid”, Voice Pathology, University of Akureyri, Akureyri, Iceland

**Keywords:** communication, experience, environment, strategies, risk factors, awareness, voice

## Abstract

**Background:**

High noise levels affect hearing, voice use, and communication. Several studies have reported high noise levels in preschools and impaired voice quality in children. Noise and poor listening conditions impair speech comprehension in children more than in adults and even more for children with hearing or language impairment, attention deficits, or another first language.

**Aim:**

The aim of this study was to explore how children in Finland, Sweden, and Iceland describe the preschool environment in relation to noise, voice, and verbal communication; what were their experiences, knowledge and ideas in relation to voice, noise, and communication. Children’s awareness of effects of noise, reactions, and coping strategies were also studied. In addition, country and gender differences were analyzed.

**Methods:**

Eighteen Icelandic, 14 Finnish, and 16 Swedish children were interviewed using a common interview-guide. Swedish and Finnish children were interviewed in focus groups and Icelandic children individually. All interviews were transcribed verbatim and analyzed thematically by the native speaker. The interviews were translated to English to be re-analyzed for inter-judge reliability of identified themes. Inter-judge reliability was calculated using percentage absolute agreement.

**Results:**

The interviews resulted in 1052 utterances, 471 from focus groups, and 581 from individual interviews. Three themes were identified, *Experiences, Environment*, and *Strategies* with two to three subcategories. Inter-judge agreement for the themes was excellent, 92–98%. Experiences occurred in 55% of the utterances. The subcategories were bodily and emotional experiences and experiences of hearing and being heard. Environment occurred in 20% of the utterances, with subcategories indoor vs. outdoor and noise. Strategies was found in 15%, with subcategories games and problem oriented actions. The only significant difference between the countries was for the theme Strategies where the Swedish children produced more utterances than the Finnish. No gender differences were found.

**Conclusion:**

Children are aware of high noise levels and mainly blame other children for making noise and shouting. They describe reactions and strategies related to noise like impaired communication and effects on hearing but are less aware of effects on voice. Expressed thoughts were similar across countries. No gender differences were found.

## Introduction

High background noise levels are well documented in preschools and schools (e.g., Sala et al., 2002; Shield and Dockrell, 2004; Sala and Rantala, 2016). Despite these environments being shared between children and adults, most studies have investigated effects of noise exposure only on teachers. Results from these studies report that high background noise levels affect general well-being (Kristiansen et al., 2013), stress (Basner et al., 2014), and of course communication and hearing (McKellin et al., 2007; Klatte et al., 2010). High background noise levels also increase vocal loudness for the speaker, known as the Lombard effect (Lane and Tranel, 1971). Increased vocal loudness increases vocal loading and reported subjective symptoms including vocal fatigue (Vilkman, 2004; Whitling et al., 2017). Long term, increased vocal loading may lead to vocal nodules (Szabo Portela et al., 2018) and impaired voice quality (Södersten et al., 2005; Ternström et al., 2006; Rantala et al., 2015; Szabo Portela et al., 2018). In preschool children, higher noise exposure also revealed an affected voice quality, with higher perceptual assessments of hoarseness, breathiness, and hyperfunction (McAllister et al., 2009). However, few studies have reported effects on children’s speech and voice in relation to different settings (e.g., Sederholm, 1995; Sederholm et al., 1995; McAllister et al., 2009; Kallvik et al., 2015). Even fewer have reported on children’s own perception of their soundscape.

In a field study of eleven 5-year old Swedish children from three preschools, voice use, and noise exposure were recorded using individually worn equipment, including two omnidirectional electret condenser microphones (TCM 110) at equal distance from the mouth and a DAT recorder. Mean background noise across children and preschools was 82.6 dB LAeq equivalent level, ranging from 81.5 to 83.6 dB LAeq for the three preschools (McAllister et al., 2009). Background noise was related to the children’s activity and peaked during lunch time, where one preschool exceeded 85 dB LAeq based on four 1 h recordings. This is alarming even if only registered during lunch time and not during the whole day. In the EU safety directives for workers, hearing protection should be provided in environments with noise levels at or above 80 dB LAeq for 8 h (European Parliament, 2003). However, preschool children are not included in the directives for workers since preschool attendance is not mandatory.

In preschools language learning, communication, and other social activities take place. For this to happen both children and adults need to be able to talk and hear each other. Studies have shown that verbal communication is hampered already at fairly low noise levels (e.g., Sala et al., 2002; Bradley and Sato, 2008). An adult person perceives approximately 95% of running speech produced at a distance of 1 m and 55 dBA background noise (ISO/TR 3352, 1974). Several studies have found that children are more impaired than adults by noisy listening conditions (Bradley and Sato, 2004; Klatte et al., 2010) and that their speech comprehension is more affected (Neuman et al., 2010). Thus, children require a better signal to noise ratio (SNR) than adults. Ratios between +6 dBA SNR and <0.5 s reverberation time (Crandell and Smaldino, 1995, 1996) to over +15 dBA SNR for the youngest children (Bradley and Sato, 2008) have been reported. Children with special needs may require even more favorable SNR and shorter reverberation times (American Speech-Language-Hearing Association, 2005). The group with special needs with regard to the needed SNR also include children with another first language (Tabri et al., 2011) since many studies have shown that non−native adult speakers have more difficulty perceiving speech in noise than native speakers (e.g., McAllister, 1990; Crandell and Smaldino, 1996; Rogers et al., 2006; Tabri et al., 2011). In a study including Swedish children learning English, Hurtig et al. (2016) reported fewer recalled words when presented in L2 compared to words presented in L1. Words presented with a high SNR (+12 dBA) improved recall compared to a low SNR (+3 dBA). Reverberation time interacted with SNR. At +12 dBA the shorter reverberation time improved recall, but at +3 dBA it impaired recall. Findings point to an increased cognitive load when perceiving L2 speech in noise. An increased cognitive load means that the listener needs to listen more attentively and that speech comprehension requires more effort. Pichora-Fuller et al. (2016) defines listening effort as “*the deliberate allocation of mental resources to overcome obstacles in goal pursuit when carrying out a task, with listening effort applying more specifically when tasks involve listening.*” Functional brain imaging reveals that the neural resources required to understand degraded speech extend beyond traditional language networks by including regions of the prefrontal cortex, premotor cortex, and the cingulo-opercular network (Peelle, 2018).

The noise in preschool is mainly activity noise. This means that the children, their speech and their activities constitute the main noise sources. According to preschool teachers, the noise levels are highest when children enter or leave the school/preschool, move from one place to another, eat lunch or play with hard toys (Jónsdóttir et al., 2015). Since children are the main noise source, they are also closer to the source compared to adults and naturally get a higher noise exposure. Adult height increases the distance to the floor and the noise source. The difference in height alone would correspond to approximately a 6 dB reduction in noise exposure, which corresponds well to reported mean noise levels at 82.6 dB LAeq in the study of child exposure (McAllister et al., 2009) and 76.1 dB LAeq based on recordings of preschool teachers (Södersten et al., 2002) using the same individually worn equipment. Speech is a strong speech masker since it has a similar spectrum as the targeted speech (Lu and Cooke, 2008). In classrooms, other student’s speech has been found to be the most disturbing noise (Boman and Enmarker, 2004).

Background noise may emanate from appliences in the building, from traffic outside the building or from the activities conducted in the building. Background noise levels caused by appliances in the school building should not exceed 28 dB LAeq or 33 dBLA_max_ according to Finnish standard (SFS 5907, 2004) but most of the classrooms – 88% – fail this target (Sala and Rantala, 2016). Effects from activity noise is harder to monitor and varies more depending on noise type. The building material may dampen or amplify sound. A building with a lot of hard surfaces contribute to increased noise by reflecting sounds in a room, thus hampering speech perception and communication. The reflections of sounds in a room are measured in terms of reverberation time. In schools and preschools, favorable listening conditions are recommended and reverberation times should be between 0.4 and 0.6 s (Crandell and Smaldino, 2000) or 0.5–0.6 s according to the Finnish standard (SFS 5907, 2004). Since children’s activities often are carried out closer to or on the floor reflections may be amplified.

Although unfavorable conditions for communication in schools and preschools are quite well documented, relatively little is known about how the children themselves perceive conditions in preschools in relation to noise, communication and voice. Interviews are frequently used to describe and explore a specific phenomenon (Malterud, 2009). Related to interviews with children fewer studies have been reported. The children’s own thoughts on their daily environment could add potentially important information to teachers, other school personnel, and builders. During the last decade there has been a growing interest of capturing this information through interviews exploring children’s own perception and reactions to road and aircraft noise (Haines et al., 2003), to noise in schools (Boman and Enmarker, 2004), noise, reactions to noise and communication in preschools (Dellve et al., 2013; Persson Waye et al., 2013) or speech disorders (Nyberg and Havstam, 2016) using individual or focus groups interviews.

The purpose of the present study was to interview preschool children from Finland, Sweden, and Iceland to increase our knowledge regarding children’s own thoughts, perception and knowledge of noise, voice, and communication. We were also interested in investigating children’s awareness of effects of noise and possible reactions to noise and to document if there were any differences between the three countries or depending on sex.

Ethical permission was obtained from the ethical board at Tampere University, Finland.

## Materials and Methods

A deductive research approach was used in the construction of the common interview guide (Williams et al., 2004). The guide included questions based on previous studies of adult voice ergonomic risk factors in learning spaces (e.g., Rantala et al., 2012), effects of noise and poor acoustics (e.g., Sala and Rantala, 2019) and also on the authors’ collective clinical and research experiences involving preschool and school aged children (Jónsdóttir, 2002; McAllister et al., 2009; McAllister, 2019; McAllister and Simberg, 2019). The questions were open-ended and wording was adapted to match children’s vocabulary and experiences (see Supplementary Appendix 1). When needed, follow-up questions and clarifications were added by the interviewer. The questions included the following main topics:

• Sounds and noise;
• Voice and perception of different voices and emotions;
• Difficulties hearing the teachers or peers and difficulties being heard by the teachers or their peers;
• Soundscape in different rooms indoors, outdoors;
• Communication related to rooms, activities and indoors vs. outdoors;
• Bodily reactions;
• Strategies used when it is noisy.

A letter about the project was sent out to the head of the preschools and when institutional participation was accepted, the teachers at the different preschools were informed. Eight preschools chose to participate, three in Finland and two each in Iceland and Sweden. An information letter was distributed to the preschools to be handed out to caregivers, and those who accepted gave a written informed consent for their child to participate. All children were age 5–6 years old, and had no known hearing, speech and language or other neurodevelopmental disorder. Eight children in the Swedish group had Swedish as their second language (L2), all other children were native speakers.

The number of children in the participating preschools were 65, 90, and 36 children in Finland (preschool 1–3, respectively), 57 and 63 (preschool 1 and 2, respectively) in Sweden and 108 and 148 (preschool 1 and 2, respectively) in Iceland. In all preschools children were divided into smaller groups of 16–21 children with three to four teachers/group. In one of the Finnish preschools, only 6-year-old children were enrolled. All other preschools had children varying from 1 to 6 years.

All preschools were situated in medium to large size university towns for the respective countries (Finland 230 000 inhabitants; Sweden 140 000, Iceland 18 000). All Finnish and Swedish preschools were runned by the city, in Iceland one was runned by the city the other was private. The preschool buildings all included at least one large gathering room and several smaller rooms for different play activities or doing arts and crafts. The outdoor play area had slides, swings, a sandbox, and a playhouse. Two Finnish preschools were on the first floor of apartment buildings and one Icelandic preschool was in a former church building. The other preschools were in buildings specifically designed for the purpose. No large roads were close to any of the preschools.

The socio-economical context of the Swedish preschools were middle class and low income, respectively with preschool 1 being in an area with below median income and preschool 2 in an area with somewhat above median income for region. The socio-economical context for the area of the Finnish and Icelandic preschools was middle class.

A total of 30 children were interviewed using focus groups with four to six children/group, 14 in Finland and 16 in Sweden. Focus group interviews of 18 children were also made in Iceland. However, the recordings were of poor quality and had to be discarded since large portions of the children’s replies could not be transcribed. The focus groups were complemented by individual interviews of 18 children from two preschools in Iceland using the same interview guide, see Table 1. All interviews were done in a separate room at the preschool. The rooms were furnished with chairs around a table to facilitate eye contact during the interviews. The children in each group knew each other well which has been found to facilitate interaction (Gill et al., 2008) and we were aiming at collecting a broad description of children’s experiences and thoughts on noise, voice and environment in the preschools. Following recommendations, especially for focus group interviews, each subject was discussed till no further comments or information were added by the children to ensure saturation in the subject (Charmaz, 2006).

**TABLE 1:** Total number of participants divided into preschools, focus groups, and individual interviews related to country. Number of boys and girls and L2 speakers are also presented.

**Country and preschools**	**Boys**	**Girls**	***n***	**No. of focus group interviews**	**No. of individual interviews**	**L2 speaker (Boys/Girls)**
Finland total	5	9	14	3		0
Preschool 1	1	3				
Preschool 2^∗^	3	3				
Preschool 3^∗^	1	4				
Sweden total	5	11	16	3		8 (3/5)
Preschool 1	0	6				
Preschool 2	5	4				
Iceland total	9	9	18		18	0
Preschool 1	3	3				
Preschool 2^∗^	6	6				
Total *N*	19	29	48	6	18	8

*^∗^Preschools not built for the purpose.*

The Finnish interviews were done with one moderator as part of a thesis project. One interviewer also carried out all the individual interviews with the children in Iceland. The Swedish focus group interviews were carried out by two moderators and speech-language pathology (SLP) students as part of their bachelor thesis. One moderator was active during the interview and one was the observer providing a second set of eyes and ears to increase the accumulation of information and to ensure validity of the analysis (Krueger and Casey, 2009). The observer handled the recording equipment and took notes during the interviews. All interviewers were certified SLPs or SLP students and all interviews were audio recorded. In Sweden a Tascam portable recorder, DR-40 and a Sennheiser microphone was used. The microphone was place on the table. In Finland a digital portable Zoom H2 recorder, with a built in microphone was used placed on the table. During the individual interviews in Iceland, an Olympus digital stereo dictaphone with a built in microphone was used and placed in front of the child. Duration of the group interviews were between 30 and 45 min and between 20 and 35 for the individual interviews.

The interviews were transcribed verbatim by the students or the native speaking author and analyzed following recommendation for qualitative content analysis (Patton, 2002; Krippendorff, 2013). This means following a step-wise procedure starting with repeated readings of the transcripts to identify meaning units (Patton, 2002; Graneheim and Lundman, 2004). The meaning units were highligted and commented in the document including first impressions and thoughts to obtain a multifacetted interpretation of the statement. Each meaning unit was condensed to reflect the main content. A single utterance could include several meaning units with different main content. In these cases utterances were split into several meaning units depending on content. The meaning units with a shared main content were grouped together. A thematic analysis of the content was made by the native speaking author and the themes were labeled to reflect included meaning units and utterances (Graneheim and Lundman, 2004). The meaning units where then further categorized into subcategories closer related to the specific topic addressed. During this process categories and themes were continuously discussed between the authors and reconsidered to ensure a trustworthy interpretation. Utterances produced by several children at the same time were excluded from the analysis since gender of the speaker could not be determined. Off topic utterances were counted but otherwise excluded since they did not contribute to the aim of the study.

All interviews were then translated to English in order to be re-analyzed by the other authors for inter-judge reliability of identified themes. All Swedish and Finnish transcriptions were re-analyzed and for the individual interviews 64% (374 utterances) were re-analyzed. Utterances categorized to a different theme were discussed between the authors to reach a final consensus. Inter-judge agreement of the thematic analyses across the three raters according to percentage absolute agreement was good to excellent varying between 92 and 98%.

### Statistics

Number of utterances across countries and related to preschool buildings were analyzed using descriptive statistics. Non-parametric statistics was used throughout. Differences in total number of utterances for each theme was analyzed using the Friedman test. Wilcoxon Signed Rank test was used for a pairwise comparison of the three themes.

Kruskal–Wallis Test was used to analyze if the distributions of utterances across themes were different between the countries and the Mann Whitney *U*-test, for independent samples, to make a pairwise comparison of the number of utterances in each theme between countries. Differences in number of produced utterances between boys and girls was analyzed using Mann Whitney *U*-test.

In all statistical analysis, *p* values < 0.05 were considered indicating significant differences.

## Results

Mean number of utterances/child depended on how talkative a child was and also on interview method, with individual interviews generating more responses/child. The mean number of utterances in the focus groups was 11.4 (SD 5.4) for Finnish children and 19.4 (SD 19.4) for Swedish. There was a difference in mean number of utterances related to preschools in the focus groups, with the children in some preschools being somewhat more talkative [Swedish preschool 1 $\bar{\text{x}}$ = 24 (SD 21.4), preschool 2 $\bar{\text{x}}$ = 16.7 (SD 18.6); Finland preschool 1 $\bar{\text{x}}$ = 15.7 (SD 7), preschool 2 $\bar{\text{x}}$ = 9 (SD 2), and preschool 3 $\bar{\text{x}}$ = 15.6 (SD 5.3)], however, the difference was not significant according to the Friedman test. For the individually interviewed Icelandic children mean number of utterances was 29.9 (SD 3.8). In the Finnish data, 10% of the children’s answers were said in unison and 8% in the Swedish. These utterances were not included in the analysis since an individual speaker could not be identified.

**FIGURE 1:**
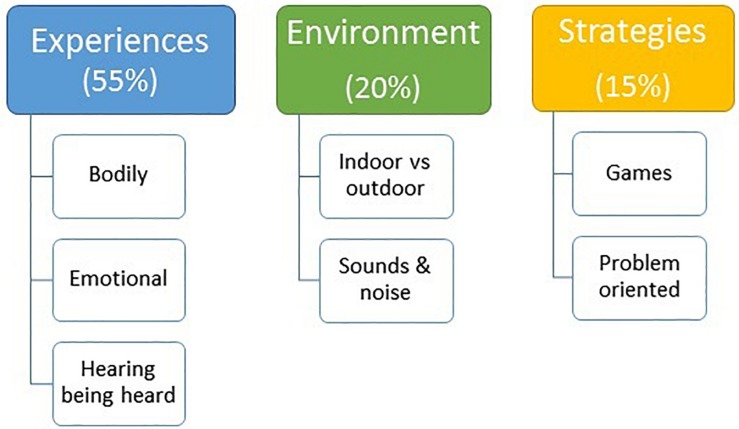
Identified themes and the percentage across all the children’s utterances and the respective subcategories/theme.

Three themes were identified, *Experiences, Environment*, and *Strategies* with two to three subcategories each, see Figure 1. Inter-judge agreement of the thematic analyses across the three raters according to percentage absolute agreement was good to excellent varying between 92 and 98%.

### Themes and Subcategories

The most common theme was related to the children’s own bodily and emotional experiences of noise, hearing and being heard themselves in the preschool. Thus *Experiences* made up a total of 55% of all utterances, followed by *Environment* and *Strategies* at 20 and 15%, respectively, see Figure 1. There was a significant difference regarding the number of utterances across themes (*p* = 0.000), with *p*-values varying from 0.004 to 0.000 in the pairwise comparisons according to the Wilcoxon Signed Rank test. Irrelevant utterances not related to the discussed topic were included in a category labeled *Other* that made up 10% of all utterances.

The number of utterances related to the preschool environment were also analyzed and compared related to the preschool buildings built for the purpose or not. In Finland there was a difference in number of utterances depending on the preschool building. Children in the preschools not original designed for the purpose commented more on the environment than other children. In Finland preschool 2 and 3 were in apartment buildings. Percentage utterances related to environment from children in preschool 2 and 3 were 19.1 and 34.3%, respectively (9 and 24 utterances) compared to only 6.7% (3 utterances) from children in preschool 1. In Iceland no such tendency was found with children in preschool 1 producing 20.9% of the utterances related to environment compared to 21.3% for children in the former church building (38 and 76 utterances, respectively). In Sweden both preschools were specifically built for the purpose. Utterances related to environment were 16.9 and 27.8%, respectively for preschool 1 and 2 (22 and 37).

**FIGURE 2:**
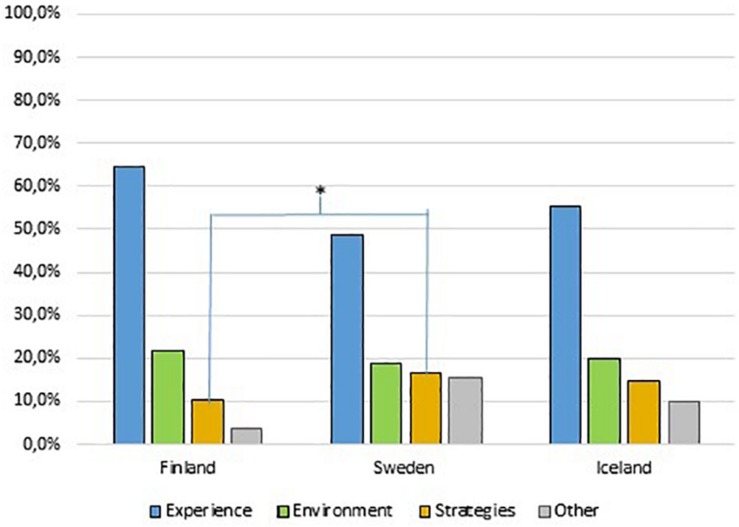
Distribution of utterances in percent across the themes for the three countries. Swedish children produced significantly more utterances related to the theme strategies compared to the Finnish children, ^∗^*p* < 0.016.

No significant difference between countries were found for the distribution of utterances across themes except for the theme Strategies, where Swedish children produced significantly more utterances than the Finnish (*p* = 0.016; see Figure 2) according to the Kruskal–Wallis Test. Regardless of country, experiences was the most common theme followed by environment and strategies, see Figure 2.

The themes could be further divided into two to three subcategories. For the theme experience they were bodily, emotional and experiences of hearing and being heard, for the theme environment subcategories were indoor vs. outdoor and sound and noise related to locality and for the theme strategies the subcategories were games and problem oriented. The distribution of responses across subcategories is shown individually for each country, see Table 2. Percentage of utterances/subcategory for each country is also presented.

**TABLE 2:** The distribution of responses across subcategories is shown individually for each country. Percentage of utterances/subcategory for each country is also presented.

		**Finland**	**Sweden**	**Iceland**
Experiences	Bodily	74 (46%)	67 (25%)	222 (42%)
	Emotional	15 (9%)	47 (18%)	22 (4%)
	Hear and being heard	18 (11%)	38 (14%)	78 (15%)
Environment	Indoor vs. outdoor	24 (15%)	32 (12%)	45 (9%)
	Sounds and noise	12 (7%)	27 (10%)	70 (13%)
Strategies	Games	1 (1%)	22 (8%)	18 (3%)
	Problem oriented	16 (10%)	30 (11%)	68 (13%)

**FIGURE 3:**
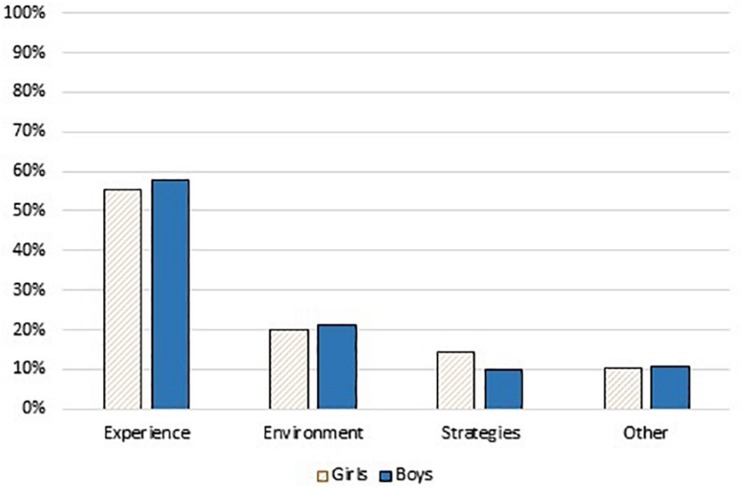
Percentage utterances by sex across the themes.

No gender differences were found related to number of produced utterances in the different themes according to the Mann Whitney *U*-test, for independent samples, see Figure 3.

Below are some examples of utterances representing the main themes and subcategories.

#### Experiences

The children connected most of the bodily and emotional experiences and reactions of voice and noise to the throat and the ears. Most children said that voice comes from the mouth or the throat but some children suggested that voice is produced in the stomach.

Examples of utterances by the children related to their bodily and emotional experience of noise, hearing and being heard are given below. Several children expressed that noise was related to when you shout and play and make “a racket.” Capital F (Finland), S (Sweden), and I (Iceland) indicates what country the child comes from. Information regarding the discussed topic is written within parenthesis. Sex of the child is indicated by G and B for girl and boy, respectively. Parentheses are added to visualize when the interviewer is asking a question, to specify the discussed topic or to provide a clarifying comment.

##### Bodily experiences

Bodily experiences and reactions were associated to noise and voice use. The children described how it felt in the body when speaking and shouting.

(F) G1: If I speak, then it kind of tickles a bit (in the mouth)…and more often when using a loud voice.
(S) G2: (Noise is) Shouting… it hurts my ears.

Some children described having a sore throat after shouting or after a day at the preschool.

(F) G1: If I shout and scream then my throat really hurts.
(S) G3: I … when I came home yesterday, I had to cough a lot, and then… then it felt like I had a lump in …
(S) G2: …the throat. (L2)

In a few cases the children also described bodily reactions related to other parts of the body like the “tummy.” In some cases these utterances were a direct response to the question: *Can you feel it somewhere in your body that you have talked too much?*

(S) B1: eeh a tingling.in my tummy.
(F) G9: I feel it all the way to my legs.

Some children associated their bodily reactions to noise like these two children describing noise being painful to their ears.

(S) G2: (Noise is) shouting… it hurts my ears.
(I) B16: Noise and pain in my ears.

A few of children expressed a more detailed anatomical and physiological knowledge about voice production, like these two children.

(F) G4: (Voice comes) from muscles that kind of start to create that voice and then here in the throat it like starts to get ready to come here and then it goes to the vocal chords and from that spot it then goes to the mouth.
(I) B16: (Voice comes) from…….the tongue…the mouth….not with the tongue as you could not talk then. From the mouth.

Seven of 18 Icelandic children (39%) said they had a sore throat sometimes when they came home from preschool and six of 16 Finnish (37%) children commented similarly. One Finnish boy (B2.2) told that he has lost his voice totally once or twice after having shouted or talked a lot. In the Swedish data four children (25%; see citations above) commented on a sore throat or a tingling in the tummy. However, the number of Swedish children having experienced a sore throat after preschool may be too low since several said they did not know what the term hoarseness or being hoarse meant. Then other children in the focus group helped explaining:

(S) B3: (it’s) like this … sss ((sounds like a snake)).

##### Emotional experiences

The children’s emotional experiences and reactions were often related to noise. Some felt bothered by the noise at the preschool and preferred the sound environment at home.

(F) B4: The noise bothers me a little bit.

The same child also mentioned that *It’s nice when it’s quiet*. This is similar to the opinion of a Swedish child (S) C5, that said *it feels good* (when it’s quiet during gathering).

(F) G9: (I do) not really (like the sounds in the day care). I like it at home in the yard.

The replies in response to what the teacher does if s/he is being firm sometimes showed that some children interpreted this as being angry.

(S) G2: she shouts instead.
(S) G8: he is angry … a little.

Although several children said that noise bothered them and that they did not feel comfortable when it was noisy there were also children that said noise did not bother them.

(F) G4: The noise doesn’t bother me at all.

##### Hearing and being heard

Several children mentioned that it sometimes was difficult both to hear others, including the teachers, and to make themselves heard at the preschool.

(I) G10: (Is it difficult for your teacher to hear you?) Yes the others are so noisy.

According to one child, a reason for the problem could be related to the teacher’s voice use and how they raised their voices.

(I) G12: they (the teachers) shout sometimes so quietly.

In spite of the shared opinion of hearing difficulties due to high noise levels, children also connected the problem with other things such as “*bad hearing.*”

(F) G1: …the ear is stuck and there’s really a lot of water in it… and then still if there’s a lot of that dirt in the ear.

Fourteen of the 18 individually interviewed Icelandic children said it was difficult to hear what the other children said and 12/18 said it was difficult to hear the teacher in the preschool. Ten of 18 said they often had to repeat themselves to be heard.

(I) G9: Yes I just say again, “thank you” and “thank you.”

#### Environment

Utterances related to different environments were mostly concerning indoors vs. outdoors and the different sound environments and noises depending on these settings. Some children also commented on specific activities or rooms and sounds in relation to that. A few utterances could be referred to both sub-categories under this theme.

##### Indoor vs. outdoor

(I) B5: Everyone talks so loud outside. Talk ordinary inside.
(S) G4: (there is more noise) inside.

The children also expressed that playing outside gave them the possibility to use their voices more freely.

(F) G9: I like that when we’re outside we scream.
(S) B1: yeah we can shout outside but talk inside.
(I) B16: Outside then very loud. You are allowed to be noisy outside.

##### Sound and noise

The children sometimes had opposite opinions on where it was most noisy.

(I) G2: There is much noise outside.
(S) G4: (there is more noise) inside.
(S) G1: (during gathering) sometimes it’s quiet like this ((just gestures)).

Several children associated noisiness with other children at the preschool. The typical comment was referring to others talking or shouting.

(F) B3: If others are talking then it’s really noisy.
(I) G10: Yes, the others are so noisy.

In addition seven of the 18 Icelandic children said they found it better to talk inside. Most Icelandic children did not find the preschool environment noisy. Common answers were: G11: *Noisy but not high*; or B16: *Sometimes (noisy) just very little.*

#### Strategies

Utterances related to different strategies involved how the children described what they or the teachers did when it was noisy, including different games or actions directly aimed at reducing noise or improving verbal communication.

##### Games

In some preschools specific games were mentioned as ways of trying to control the noise.

(S) B4: sometimes we play the silent game.

In Swedish and Icelandic preschools children described a strategy to lower the noise level related to different activities involving whispering. From the childrens comments this strategy seemed to be used most often during lunchtime.

(S) B1: then you have to whisper And then we have to whisper…and the table that whispers the least wins and they they become whispermasters… today me and my best friend will be whispermasters.
(S) G5: during the silent game you have to be completely quiet but during whisper-lunch you have to whisper.
(S) G2: sometimes we sing doing signs.

In Iceland it was common for the teachers to ask the children to use their “*indoor voice*” when they were being too loud, according to some comments.

##### Problem oriented

Problem oriented strategies were actions directly aimed at an undesired behavior, improving communication or ways to avoid noise exposure. Typical strategies were to cover your ears or comments related to how you improve verbal communication.

(S) B3: if you do like this ((covers the ears)).
(S) B4: cause you cover your ears.
(F) G2: …if someone is speaking, you don’t talk over him/her and zip it (mouth).
(F) B5: If someone shouts then you must hush him/her.
(I) B7: By stopping talking so much.

One child had realized herself that she could rest her voice by taking a pause from speaking.

(F) G5: Often if I run really fast or speak really loud then I start to feel like I should stop for a while.

Another strategy could be to improve your own speech by talking loud and clear so others can hear you.

(I) G12: Just talk loud and clear.

In one Finnish preschool a traffic-light system also including a warning sound, had been implemented to alert everybody when noise levels were too high. The red light and the sound meant that you needed to lower your vocal loudness and be more quiet.

(F) G4: …there’re those kinds of traffic lights and you hear choo-choo-choo and then the red light turns on and it means that you gotta lower your voice.

In some cases the children expressed that the thing to do when it was noisy was to get a teacher that could calm the noisy children.

(S) G5: yah but we do it anyway (shout), I know one time, if, if someone wanted to tell the teachers that they should come and say something (to the noisy children).

#### Other

The category other was mostly made up by utterances that were regarded as irrelevant and off topic. However, in some cases, especially among the Swedish L2 speakers, there were also clear misunderstandings. Below are two examples.

(S) G1: (Noise is) it’s like eating (L2) (misunderstands buller as bullar, in English buns).
(S) G9: (Noise is) when you bake (L2).
(S) G1: (Voice is) that you should vote (L2) (misunderstands röst as rösta, in English vote).

Some Finnish children talked about “sound” instead of “voice” because the same word is used for both these concepts. In addition, two children talked about difficulties to understand foreign language when the topic of discussion was about difficulties hearing what the teachers’ said.

In the individual interviews there were significantly more *I don’t know* replies compared to the group interviews, in total 39 from the Icelandic children compared to two and one, respectively, in the Finnish and Swedish groups (*p* = 0.004 and 0.001, respectively) according to a Mann Whitney *U*-test, for independent samples. One Icelandic child contributed with 12 *I don’t know* replies. A higher number can be expected in the individual interviews since all children needed to respond to all questions where in the focus groups those who felt sure about the concept replied. Twelve Icelandic children replied “*I don’t know*” between 1 and 12 times. Mean was 2.2 times (SD 3.03).

## Discussion

In this study a total of 48 children were interviewed, in focus groups or individually in their preschools. The results show that the children’s comments on sound and communication in the preschool was related to their own personal experiences of what they had seen, heard and felt. The results also revealed a budding awareness of high noise levels in the preschool and by describing effects on hearing and communication as well as strategies to avoid or decrease noise exposure. The children mostly described themselves or other children as the main noise source. Several children blamed the noise on other children playing and shouting. The children were less aware of effects of noise on voice but some had experienced a sore throat after preschool. Our findings are very similar to those of Dellve et al. (2013) also including focus group interviews of Swedish preschool children.

In everyday conversations, hearing is synonymous with understanding the content of what was said. This is also how the term to hear was interpreted in the interviews. However, when you hear a speaker talking a foreign language you can hear perfectly well but still not understand the content of the speech. This ambiguity of how the term is used among laymen was illustrated in some of the interviews, where the Icelandic children sometimes confused difficulties to hear what was said with difficulties understanding the content of the utterance and Finnish children who mentioned difficulties to understand foreign languages when discussing difficulties hearing what the teachers’ said.

The most commonly identified theme in the children’s utterances was related to the children’s *Experiences* of noise and difficulties hearing others and being heard by others as well as bodily and emotional experiences. For bodily reactions most children talked about ears hurting or a sore throat related to noise and loud voice use. However, also other body parts were mentioned like tingling in the mouth, tummy, or even legs. This is similar findings reported by Persson Waye et al. (2013). They interpreted the comments as a tendency for children to describe reactions to noise in a somatic way directly felt in the body (head, tummy) compared to adults. Another common response under this theme was that the children had problems hearing the teacher or other children. They also expressed that they sometimes had to shout and repeat what they said to make themselves heard and that the teachers shouted at times for the same reason. Most children in the individual interviews said they had experienced difficulties both hearing other children, the teachers and making themselves heard. Thus, the children have a potential awareness of effects of noise on communication. In a previous study on teachers use of amplification (WL 184 lapel condenser chest microphone combined with an amplifier and portable loudspeaker) over 95% of the participating children (6–9 years old) said that the use of amplification facilitated listening (“*I can hear better*”). They also asserted that “*the teacher does not shout as much*” and “*she is not so angry*” (Jónsdóttir, 2009). In the present study the children found it difficult to interpret the emotion of a teacher with a loud voice. In a noisy environment a loud voice is often necessary to make yourself heard. For the children a teacher’s loud voice was often interpreted as angry. Brännström et al. (2015) reported similar findings that emotional content was more difficult both to convey and perceive in a noisy environment probably due to effects related to vocal loudness.

The children mostly blamed others than themselves for making noise and shouting. This shows an awareness of negative effects of noisy environments and that children, due to this awareness, they don’t want to be blamed for being noise-makers. Like an Icelandic boy said, B18: *Yes sometimes I make noise just by accident*. This statement is well in line with what we all do automatically in a noisy setting, we increase vocal loudness (the Lombard effect; Lane and Tranel, 1971). Blaming other children for being noisy may also indicate that that listening conditions were generally unfavorable or that the high sound pressure levels impaired hearing and speech comprehension, possibly affecting the SNR required for good listening conditions for young children (Neuman et al., 2010). In the studied age-group children may need over +15 dBA SNR for good speech comprehension (Bradley and Sato, 2008) and children with special needs probably even more favorable conditions (American Speech-Language-Hearing Association, 2005). This includes children with another first language (Tabri et al., 2011). During the interviews two of the children with Swedish as their second language misunderstood phonetically similar words. Such misunderstandings were not found in the children speaking their first language. However, the larger number of *I don’t know* replies in the individual interviews may also reflect a lack of understanding the questions or the terms used. The noisy conditions in the preschool could delay speech development and speech understanding due to the required SNR for good speech comprehension. Here also native speaking children may be at risk due to the frequent comment about difficulties both hearing others and making themselves heard. In the long-term this may affect vocabulary and later reading and writing skills (Shield and Dockrell, 2008). In school children indoor noise and reverberation in classrooms were found to be associated with poorer performance in verbal tasks (Klatte et al., 2013). The findings point to the importance of good listening conditions for language learning and communication in children in general and especially for L2 speakers.

It was common to express a relation between noise and shouting but these utterances did not connect noise and shouting to having a sore throat. The habitual use of a loud voice during preschool hours may adapt children to this increased vocal loudness. On the basis of our clinical experience, parents often describe their child’s speaking voice as being very loud when leaving preschool. This adaptation has been found also in teachers, where teachers working in loud background noise used louder voices already in the morning before work compared to those with classes with lower noise levels (Rantala et al., 2015). It seems reasonable to assume that vocal habits are established during childhood. Thus, undesirable and potentially straining speaking styles established during childhood may continue into adult life. The long term effects of maintaining a loud voice have not, as far as we are aware, been well documented scientifically even if several studies indicate a relationship between several vocal symptoms and vocally demanding professions (e.g., Fritzell, 1996; Roy et al., 2004). In the present study children sometimes commented on not being able to hear the teachers and, a few times, also on the teachers voices; (I) G12: *they* (the teachers) *shout sometimes so quietly.* The comment may indicate that this teacher had vocal fatigue and was unable to raise vocal loudness. Sala et al. (2001) found a strong association between the teaching profession and the 12 months prevalence of vocal fatigue. In a study on recovery after short term vocal loading in adults, patients with functional dysphonia were found to have slower recovery than the controls (Whitling et al., 2017). The recovery time for children after vocal loading, for example that during a day in a noisy preschool, has not been studied systematically. However, perceptual differences have been reported when comparing morning and afternoon recordings from children in preschool (McAllister et al., 2009) and in school children who had attended preschool and after school care compared with those who had not (Sederholm et al., 1995). This could imply a habituation to loud voice use as a long term effect of attending preschool.

The three themes *Experiences, Environment*, and *Strategies* were found in all interviews and no significant differences were found regarding the number of utterances between the three countries or depending on interview method except in the theme Strategies where the Swedish children had significantly more utterances than the Finnish. However, despite this the strategies to control noise by games and other actions that emerged between the two countries were very similar. Thus, the different rate of utterances regarding strategies was probably incidental. The off topic utterances were collected under the label other. Here utterances not connected to the discussion or misunderstandings were placed. The topics brought up included ballet dancing, clothes and traveling but has not been further analyzed.

The strategies the children describe were mostly related to different actions intended to lower the noise levels. The “silent game” and the “whisper lunch” both aim at this. The whisper lunch may do just that but to whisper is also quit a straining speaking technique that should be used with some caution. Other utterances included to *go get the teacher* when it was too noisy or describing that you need to *be quiet yourself* or *tell others to quiet down* to be able to listen properly. Some children described how to avoid noise by *covering their ears*. This is similar to findings by Dellve et al. (2013) also using focus group interviews. The children were also able to compare different environments with varying amounts of noise. These comparisons usually involved the preschool and the home environment *(I do) not really (like the sounds in the day care). I like it at home in the yard.* Several children felt bothered by the noise and preferred when it was quiet. Still, some said that noise did not bother them.

In a recently published study including children 9–13 years, findings suggested that noise conditions in crowded spaces are most challenging (Brännström et al., 2017). They also found that the extent of annoyance caused by the noise was task dependent, with tasks with high demands on verbal processing being more affected. Based on the noise mentioned most often in the present study, other children playing or shouting and teachers shouting seems to be the most disturbing noise for preschool children. This typically takes place in crowded spaces like the lunch room or the play hall. The reported annoyance related to verbal processing could also be linked the difficulties hearing others that was often described by the preschool children in the present study.

The children had more knowledge about noise and communication than we expected. They were aware that noise affects hearing and expressed difficulties both hearing others and making themselves heard. However, the connection between speaking in a noisy environment and having a soare throat was generally not made. This seems to reflect a knowledge gap regarding the potentially harmful effects of speaking in noise. Although the children were able to reflect on their preschool sound environment, some, especially the individually interviewed Icelandic children, responded “*I don’t know*” quite often. Often these replies were following questions on voice or voice use. These replies might mirror a lack of knowledge or an insecurity about the topic. A higher number of such replies could be expected in the individual interviews since all children needed to respond to all questions even if they were not sure. In the focus groups the total number of these replies was one or two, respectively for Sweden and Finland.

### Methodological Considerations

The interview guide was designed based on previous studies of adults (e.g., Rantala et al., 2012; Sala and Rantala, 2019) also including the authors’ collective clinical and research experience of studies involving children (Jónsdóttir, 2002; McAllister et al., 2009; McAllister, 2019; McAllister and Simberg, 2019). The questions were adapted to children by using a simpler language and, if needed, terminology was explained further. Most questions were open ended in order to provide longer responses and start a discussion among the children but a few were direct dicotomic yes/no questions. However, children mostly answered with longer utterances also to these questions showing an unexpected competence and ability to reflect.

The focus group interviews and the individual interviews provided an extensive material that allowed the children themselves to voice their opinions, perception, and knowledge on noise, voice and communication. Since they all knew each other well most children participated and contributed to the group interviews but the number of utterances varied with one or two children in each group contributing only a limited number. The individual interviews were included to amend the possible effect of more talkative and outgoing children’s opinions that could dominate responses in the focus groups.

In the group interviews, the children sometimes had a tendency to repeat what another child had just uttered. This phenomenon called “other repetition” does not always mean that a child just imitates the friend. It has been shown that these repetitions have several functions in children’s conversational discourses, such as affirming, agreeing with the other speaker, making matching and counter-claims (Keenan, 1975; Huang, 2010). Other repetition is very typical among preschool children while talking together (Karjalainen, 1996). In our study, some children’s opinions undoubtedly were adopted from their peers’. Still, these utterances seemed to reflect also the repeating child’s own perceptions and views.

The effects of the different interview methods on the results, if any, are difficult to assess. No differences were found related to interview method apart from significantly more “I don’t know” replies given during the individual interviews. Nonetheless, the individual interviews confirmed observations in the focus groups and added information regarding how many children this applied to. One such example is how Icelandic children mentioned difficulties both hearing others (13/18), including the teachers (12/18), and being heard themselves (14/18). They also mentioned that they often had to repeat themselves (10/18). This was also found in the focus groups but how common it was could not be established.

There were no measurements made of the participating preschools regarding noise levels with children present or empty, nor regarding reverberation times. Both these measures would have added potentially important information on background noise and acoustic properties of the preschools but this was not the focus of the present study. Two included preschools were located in apartment buildings. There was a clear tendency for children in these preschools to talk about the environment more than other children. None of the included children had special needs known to the parents or preschool teachers at the time of the interviews. In the Swedish group, eight children with Swedish as a second language were included. More children with Swedish, Finnish or Icelandic as their second language could have added information regarding their specific difficulties in a noisy setting.

### Practical Implications and Future Studies

Practical implications of the present results are the need of an increased awareness and knowledge regarding the effects of noise in preschools. The findings also point to a knowledge gap regarding how high noise levels affect voice use. Considering children’s learning potential and curiosity, an adapted education material for preschool children is surely needed. In future studies it would be interesting to study pedagogical effects on noise and communication and to include more L2 speakers to study possible effects of noise on language learning, vocabulary and comprehension using focus groups. It would also be interesting to interview other potentially vulnerable groups such as children with language disorders, attention deficits or cognitive impairment to study their thoughts, comprehension and reactions to noise and effects on communication. The differences in number of responses from children in preschools in apartment buildings may point to a need to study effects of environment on preschool children in more detail. Several factors may contribute since figures varied also between children in preschools built for the purpose.

## Conclusion

Children are aware of high noise levels and blame other children for making noise and shouting. They describe reactions and strategies related to noise. They are aware of impaired communication in noise and effects on hearing but less aware of effects on voice. The experiences of children from three Nordic countries are quite similar possibly reflecting a shared cultural background. In addition, girls and boys describe their preschool sound environment and difficulties related to communication alike.

## Data Availability

The datasets generated for this study are available on request to the corresponding author.

## Ethics Statement

Ethical permission was obtained from the ethical board at Tampere University, Finland. A letter about the project was sent to the head of preschools and when institutional participation was accepted, the teachers at the different preschools were informed. Seven preschools chose to participate, three in Finland and two each in Iceland and Sweden. An information letter was distributed to the preschools to be handed out to caregivers, and those who accepted gave a written informed consent for their child to participate.

## Author Contributions

All authors contributed to the common interview guide and the data collection in collaboration with SLP-students, Erica Domej, Malin Eriksson, and Liisa Petäjistö, and with SLP Gudrun Sigurdardottir, analyzed the data, and discussed and provided the comments on the manuscript. Statistical analysis was mainly carried out by LR with the support of AM. Manuscript writing was mainly carried out by AM with the support of LR.

## Conflict of Interest Statement

The authors declare that the research was conducted in the absence of any commercial or financial relationships that could be construed as a potential conflict of interest.

## References

[B1] American Speech-Language-Hearing Association. (2005). *Guidelines for Addressing Acoustics in Educational Settings.* Rockville, MD: American Speech-Language-Hearing Association.

[B2] Basner M. Babisch W. Davis A. Brink M. Clark C. Janssen S. (2014). Auditory and non-auditory effects of noise on health. *Lancet* 383 1325–1332. 10.1016/s0140-6736(13)61613-x 24183105 PMC3988259

[B3] Boman E. Enmarker I. (2004). Factors affecting pupils noise in schools: the building and testing models. *Environ. Behav.* 36 207–228. 10.1177/001391650325664

[B4] Bradley J. S. Sato H. (2004). Speech recognition by grades 1, 3 and children in classrooms. *Can. Acoust.* 32 26–27.

[B5] Bradley J. S. Sato H. (2008). The intelligibility of speech in elementary school classrooms. *J. Acoust. Soc. Am.* 123 2078–2086. 10.1121/1.2839285 18397015

[B6] Brännström K. J. Holm L. Lyberg-Åhlander V. Haake M. Kastberg T. Sahlén B. (2015). Children’s subjective ratings and opinions of typical and dysphonic voice after performing a language comprehension task in background noise. *J. Voice* 29 624–630. 10.1016/j.jvoice.2014.11.003 25873548

[B7] Brännström K. J. Johansson E. Vigertsson D. Morris D. J. Sahlén B. Lyberg-Åhlander V. (2017). How children perceive the acoustic environment of their school. *Noise Health* 19 84–94. 10.4103/nah.NAH_33_16 29192618 PMC5437757

[B8] Charmaz K. (2006). *Constructing Grounded Theory: A Practical Guide Through Qualitative Analysis.* Thousand Oaks, CA: Sage Publications.

[B9] Crandell C. C. Smaldino J. (1995). The effects of room acoustics on normal-hearing children: implications for intervention. *J. Acoust. Soc. Am.* 97 3262–3262. 10.1121/1.411633

[B10] Crandell C. C. Smaldino J. J. (1996). Speech perception in noise by children for whom english is a second language. *Am. J. Audiol.* 5 47–51. 10.1044/1059-0889.0503.47

[B11] Crandell C. C. Smaldino J. J. (2000). Classroom acoustics for children with normal hearing and with hearing impairment. *Lang. Speech Hear. Serv. Sch.* 31 362–370. 10.1044/0161-1461.3104.362 27764475

[B12] Dellve L. Samuelsson L. Persson Waye K. (2013). Preschool children’s experience and understanding of their soundscape. *Qual. Res. Psychol.* 10 1–13. 10.1080/14780887.2011.586099

[B13] European Parliament. (2003). Directive 2003/10/EC on the minimum health and safety requirements regarding exposure of workers to the risk arising from physical agents (noise). *OJEC* L 042, 38–44.

[B14] Fritzell B. (1996). Voice disorders and occupations. *Logop. Phoniatr. Voco.* 2 7–12. 10.3109/14015439609099197

[B15] Gill P. Stewart K. Treasure E. Chadwick B. (2008). Methods of data collection in qualitative research: interviews and focus groups. *Br. Dent. J.* 204 291–295. 10.1038/bdj.2008.192 18356873

[B16] Graneheim U. H. Lundman B. (2004). Qualitative content analysis in nursing research: concepts, procedures and measures to achieve trustworthiness. *Nurse Educ. Today* 24 105–112. 10.1016/j.nedt.2003.10.001 14769454

[B17] Haines M. M. Brentnall S. L. Stansfeld S. A. Klineberg E. (2003). Qualitative responses of children to environmental noise. *Noise Health* 5 19–30. 12804209

[B18] Huang C. C. (2010). Other-repetition in Mandarin child language: a discourse-pragmatic perspective. *J. Pragmat.* 42 825–839. 10.1016/j.pragma.2009.08.005

[B19] Hurtig A. Keus van de Poll M. Pekkola E. P. Hygge S. Ljung R. Sörqvist P. (2016). Children’s recall of words spoken in their first and second language: effects of signal-to-noise ratio and reverberation time. *Front. Psychol.* 6:2029. 10.3389/fpsyg.2015.02029 26834665 PMC4712295

[B20] ISO/TR 3352 (1974). *Acoustics – Assessment of Noise with Respect to its Effect on the Intelligibility of Speech.* Switzerland: International Organization for Standardization.

[B21] Jónsdóttir V. (2009). *Is Amplification Necessary in a Classroom?.* Lissabon: Internoise.

[B22] Jónsdóttir V. Rantala L. M. Oskarsson G. K. Sala E. (2015). Effects of pedagogical ideology on the perceived loudness and noise levels in preschools. *Noise Health* 17 282–293. 10.4103/1463-1741.165044 26356370 PMC4900493

[B23] Jónsdóttir V. I. (2002). Cordless amplifying system in classrooms. A descriptive study of teachers’ and students’ opinions. *Logop. Phoniatr. Voco.* 27 29–36. 10.1080/140154302760146952 12375626

[B24] Kallvik E. Lindström E. Holmqvist S. Lindman J. Simberg S. (2015). Prevalence of hoarseness in school-aged children. *J. Voice* 29 260.e1–260.e19. 10.1016/j.jvoice.2013.08.019 25017976

[B25] Karjalainen M. (1996). *There is a Monster Here, in Play. Analysis of Three- and Four-Year-Old Preschool Children‘s Conversations in Terms of Acts, Turns, Topics and Special Features.* Dissertation, University of Oulu: Oulu.

[B26] Keenan E. O. (1975). Making it last: repetition in children’s discourse. *Annu. Meet. Berkeley Ling. Soc.* 1 279–294. 10.3765/bls.v1i0.2336

[B27] Klatte M. Bergström K. Lachmann T. (2013). Does noise affect learning? A short review on noise effects on cognitive performance in children. *Front. Psychol.* 4:578. 10.3389/fpsyg.2013.00578 24009598 PMC3757288

[B28] Klatte M. Lachmann T. Meis M. (2010). Effects of noise and reverberation on speech perception and listening comprehension of children and adults in a classroom-like setting. *Noise Health* 12 270–282. 10.4103/1463-1741.70506 20871182

[B29] Krippendorff K. (2013). *Content Analysis: An Introduction to Its Methodology.* Thousand Oaks, CA: SAGE Publications.

[B30] Kristiansen J. Persson R. Lund S. P. Shibuya H. Nielsen P. M. (2013). Effects of classroom acoustics and self-reported noise exposure on teachers’ well-being. *Environ. Behav.* 45 283–300. 10.1177/0013916511429700

[B31] Krueger R. Casey M. A. (2009). *Focus Groups. A Practical Guide for Applied Research.* Thousand Oaks, CA: Sage Publications.

[B32] Lane H. Tranel B. (1971). The Lombard sign and the role of hearing in speech. *J. Speech Hear. Res.* 14 677–709. 10.1044/jshr.1404.677

[B33] Lu Y. Cooke M. (2008). Speech production modifications produced by competing talkers, babble, and stationary noise. *J. Acoust. Soc. Am.* 124 3261–3275. 10.1121/1.2990705 19045809

[B34] Malterud K. (2009). *Qualitative Methods in Medical Research [in Swedish].* Lund: Studentlitteratur.

[B35] McAllister A. (2019). “Voice ergonomics for children. Children’s voice ergonomics,” in *Voice Ergonomics. Occupational and Professional Voice Care*, eds Sala E. Rantala L., (Newcastle upon Tyne: Cambridge Scholars Publishing), 130–153.

[B36] McAllister A. Granqvist S. Sjölander P. Sundberg J. (2009). Child voice and noise: a pilot study of the effect of a day at the day-care on ten children’s voice quality according to perceptual evaluation. *J. Voice* 23 587–593. 10.1016/j.jvoice.2007.10.017 18456454

[B37] McAllister A. Simberg S. (2019). “Children’s voice ergonomics,” in *Voice Ergonomics. Occupational and Professional Voice Care*, eds Sala E. Rantala L., (Newcastle upon Tyne: Cambridge Scholars Publishing), 221–224.

[B38] McAllister R. (1990). *Perceptual Foreign Accent: l2 Users’ Comprehension Ability. New Sounds 90.* Amsterdam: University of Amsterdam.

[B39] McKellin W. H. Shahin K. Hodgson M. Jamieson J. Pichora-Fuller K. (2007). Pragmatics of conversation and communication in noisy settings. *J. Pragmat.* 39 2159–2184. 10.1016/j.pragma.2006.11.012

[B40] Neuman A. C. Wroblewski M. Hajicek J. Rubinstein A. (2010). Combined effects of noise and reverberation on speech recognition performance of normal-hearing children and adults. *Ear Hear.* 31 336–344. 10.1097/AUD.0b013e3181d3d514 20215967

[B41] Nyberg J. Havstam C. (2016). Speech in 10-year-olds born with cleft lip and palate: what do peers say? *Cleft Palate Craniofac. J.* 53 516–526. 10.1597/15-140 26418146

[B42] Patton M. (2002). *Qualitative Research & Evaluation Methods*, 3rd ed Thousand Oaks, CA: SAGE Publication.

[B43] Peelle J. E. (2018). Listening effort: how the cognitive consequences of acoustic challenge are reflected in brain and behavior. *Ear Hear.* 39 204–214. 10.1097/AUD.0000000000000494 28938250 PMC5821557

[B44] Persson Waye K. van Kamp I. Dellve L. (2013). Validation of a questionnaire measuring preschool children’s reactions to and coping with noise in a repeated measurement design. *BMJ Open* 3:e002408. 10.1136/bmjopen-2012-002408 23793676 PMC3657644

[B45] Pichora-Fuller M. K. Kramer S. E. Eckert M. A. Edwards B. Hornsby B. W. Humes L. E. (2016). Hearing impairment and cognitive energy: the framework for understanding effortful listening (FUEL). *Ear Hear.* 37 5S–27S. 10.1097/aud.0000000000000312 27355771

[B46] Rantala L. M. Hakala S. Holmqvist S. Sala E. (2015). Classroom noise and teachers’ voice production. *J. Speech Lang. Hear. Res.* 58 1397–1406. 10.1044/2015_JSLHR-S-14-0248 26089145

[B47] Rantala L. M. Hakala S. J. Holmqvist S. Sala E. (2012). Connections between voice ergonomic risk factors and voice symptoms, voice handicap, and respiratory tract diseases. *J. Voice* 26:819.e13-20. 10.1016/j.jvoice.2012.06.001 23044460

[B48] Rogers C. L. Lister J. L. Febo D. M. Besing J. M. Abrams H. B. (2006). Effects of bilingualism, noise, and reverberation on speech perception by listeners with normal hearing. *Appl. Psycholinguist.* 27 465–485. 10.3766/jaaa.15125 28534731

[B49] Roy N. Merrill R. M. Thibeault S. Parsa R. A. Gray S. D. Smith E. M. (2004). Prevalence of voice disorders in teachers and the general population. *J. Speech Lang. Hear. Res.* 47 281–293. 15157130 10.1044/1092-4388(2004/023)

[B50] Sala E. Airo E. Olkinuora P. Simberg S. Ström U. Laine A. (2002). Vocal loading among day care center teachers. *Logop. Phoniatr. Voco.* 27 21–28. 10.1080/140154302760146943 12375625

[B51] Sala E. Laine A. Simberg S. Pentti J. Suonpaa J. (2001). The prevalence of voice disorders among day care center teachers compared with nurses: a questionnaire and clinical study. *J. Voice* 15 413–423. 10.1016/s0892-1997(01)00042-x 11575637

[B52] Sala E. Rantala L. (2016). Acoustics and activity noise in school classrooms in Finland. *Appl. Acoust.* 114 252–259. 10.1016/j.apacoust.2016.08.009

[B53] Sala E. Rantala L. (2019). “Noise and room acoustics,” in *Voice Ergonomics. Occupational and Professional Voice Care*, eds Sala E. Rantala L., (Newcastle upon Tyne: Cambridge Scholars Publishing), 172–178.

[B54] Sederholm E. (1995). Prevalence of hoarseness in ten-year-old children. *Scand. J. Logop. Phoniatr.* 20 165–173. 10.3109/14015439509098744 8563778

[B55] Sederholm E. McAllister A. Dalkvist J. Sundberg J. (1995). Aetiologic factors associated with hoarseness in ten-year-old children. *Folia Phoniatr. Log.* 5 262S–278S. 8563778 10.1159/000266360

[B56] SFS 5907 (2004). *Acoustic Classification of Spaces of Buildings.* Helsinki: Finnish Standards Association.

[B57] Shield B. Dockrell J. E. (2004). External and internal noise surveys of London primary schools. *J. Acoust. Soc. Am.* 115 730–738. 10.1121/1.1635837 15000185

[B58] Shield B. M. Dockrell J. E. (2008). The effects of environmental and classroom noise on the academic attainments of primary school children. *J. Acoust. Soc. Am.* 123 133–144. 10.1121/1.2812596 18177145

[B59] Södersten M. Grankvist S. Hammarberg B. Szabo A. (2002). Vocal behavior and vocal loading factors for preschool teachers at work studied with binaural DAT recordings. *J. Voice* 16 356–371. 10.1016/s0892-1997(02)00107-8 12395988

[B60] Södersten M. Ternström S. Bohman M. (2005). Loud speech in realistic environmental noise: phonetogram data, perceptual voice quality, subjective ratings, and gender differences in healthy speakers. *J. Voice* 19 29–46. 10.1016/j.jvoice.2004.05.002 15766848

[B61] Szabo Portela A. Granqvist S. Ternström S. Södersten M. (2018). Vocal behavior in environmental noise: comparisons between work and leisure conditions in women with work-related voice disorders and matched controls. *J. Voice* 32:126.e23-e38. 10.1016/j.jvoice.2017.04.010 28551331

[B62] Tabri D. Abou Chacra K. M. Pring T. (2011). Speech perception in noise by monolingual, bilingual and trilingual listeners. *Int. J. Lang. Commun. Disord.* 46 411–422. 10.3109/13682822.2010.519372 21771217

[B63] Ternström S. Bohman M. Södersten M. (2006). Loud speech over noise: some spectral attributes, with gender differences. *J. Acoust. Soc. Am.* 119 1648–1665. 10.1121/1.2161435 16583909

[B64] Vilkman E. (2004). Occupational safety and health aspects of voice and speech professions. *Folia Phoniatr. Logop.* 56 220–253. 10.1159/000078344 15258436

[B65] Whitling S. Lyberg-Åhlander V. Rydell R. (2017). Recovery from heavy vocal loading in women with different degrees of functional voice problems. *J. Voice* 31 .e1–.e645. 10.1016/j.jvoice.2016.12.012 28572014

[B66] Williams C. Bower E. J. Newton J. T. (2004). Research in primary dental care part 6: data analysis. *Br. Dent. J.* 197 67–73. 10.1038/sj.bdj.4811467 15272337

