# Supplementary material for: The Others Are Too Loud! Children’s Experiences and Thoughts Related to Voice, Noise, and Communication in Nordic Preschools
Source: Front Psychol. 2019 Aug 21;10:1954. doi: 10.3389/fpsyg.2019.01954 (PMC6712832; doi:10.3389/fpsyg.2019.01954)
Supplement: Supplementary file 1 [file Data_Sheet_1.docx]

Appendix

Questions asked children.

| Aim of the topic: | Questions asked | Additional questions when needed |
| --- | --- | --- |
| Understand concepts | What do you think (1) sound, (2) silence, (3) voice, (4) noise is? | Can you make sounds? |
| Voice | Where does the voice come from? |  |
|  | How does your voice feel*?* | Can you feel it somewhere in your body? Where do you feel it? |
|  | What kind of voice do you like/dislike? | Ask children to imitate a pleasant/ unpleasant voice.  Can you feel it somewhere in your body? Where do you feel it? |
| Hearing | What kind of situations can your teachers and your friends hear you best? They find it hard to hear you? | When? Where? Why do you think that is? |
|  | What kind of situations can you hear your teachers and your friends best? You cannot hear them? | When? Where? Why do you think that is?  What do you think what could be done to make it better? |
| Sound en­­vironment | How does it sound inside in your preschool? | In a cushion room? Lunch room? Gathering room? Entrance hall?  Is it different when it comes to talking in these rooms? |
|  | How does it sound outside in your preschool playground? | Is talking different outside or inside? How? |
|  | When do you talk the most? | While eating? While playing? |
|  | Do you have to repeat yourself sometimes**?** | When? Where? Why do you think that is? |
|  | Does it happen that you have to scream sometimes to be heard? | When? Where? Why do you think that is? |
| Strategies to lower noise levels | Do you have to be quiet sometimes? | Does it happen that your teacher says ”schhh”? Why? Do you sometimes play games where you have to be quiet? When? What game? |
| Vocal health | Do you sometimes have a sore throat? | When, why? |
